# Gene Interaction Network Analysis Reveals IFI44L as a Drug Target in Rheumatoid Arthritis and Periodontitis

**DOI:** 10.3390/molecules27092749

**Published:** 2022-04-25

**Authors:** Pradeep Kumar Yadalam, Thilagar Sivasankari, Santhiya Rengaraj, Maryam H. Mugri, Mohammed Sayed, Samar Saeed Khan, Mona Awad Kamil, Shilpa Bhandi, A. Thirumal Raj, Shankargouda Patil, Artak Heboyan

**Affiliations:** 1 Department of Periodontics, Saveetha Dental College and Hospitals, Saveetha Institute of Medical and Technical Sciences, Saveetha University, Chennai 600077, India; pradeepkumar.sdc@saveetha.com; 2 Department of Periodontology, Adhiparasakthi Dental College and Hospital, Melmaruvathur 603319, India; drsivasankarit@apdch.edu.in (T.S.); santhiyarengaraj96@gmail.com (S.R.); 3 Department of Maxillofacial Surgery and Diagnostic Sciences, College of Dentistry, Jazan University, Jazan 45412, Saudi Arabia; dr.mugri@gmail.com; 4 Department of Prosthetic Dental Sciences, College of Dentistry, Jazan University, Jazan 45412, Saudi Arabia; drsayed203@gmail.com; 5 Department of Maxillofacial Surgery and Diagnostic Sciences, Division of Oral Pathology, College of Dentistry, Jazan University, Jazan 45142, Saudi Arabia; samarkhan8@gmail.com; 6 Department of Preventive Dental Science, College of Dentistry, Jazan University, Jazan 45412, Saudi Arabia; munakamil@yahoo.com; 7 Department of Restorative Dental Science, College of Dentistry, Jazan University, Jazan 45142, Saudi Arabia; shilpa.bhandi@gmail.com; 8 Department of Oral Pathology and Microbiology, Sri Venkateswara Dental College and Hospital, Chennai 600130, India; thirumalraj666@gmail.com; 9 Department of Prosthodontics, Faculty of Stomatology, Yerevan State Medical University after Mkhitar Heratsi, Str. Koryun 2, Yerevan 0025, Armenia

**Keywords:** periodontitis, drug target, molecular docking, rheumatoid arthritis, vemurafenib

## Abstract

**Simple Summary:**

In spite of substantial investigation, the biological link between periodontitis and rheumatoid arthritis remains unexplained. This study intended to correlate periodontitis and rheumatoid arthritis gene expression patterns to find shared targets for both the disease. We identified the differentially expressed genes (DEGs) in periodontitis and rheumatoid arthritis. The network was built by integrating DEGs and ranking the genes using GeneMANIA. FINDSITEcomb2.0 was used to find a possible inhibitor for the top-ranked gene. Further, the binding effectiveness and protein-ligand complex stability were then determined by molecular docking and molecular dynamics. The network analysis showed IFI44L as a highly ranking gene implicated in most immunological pathways. A virtual screening of 6507 compounds revealed vemurafenib as the best candidate for the IFI44L target. Molecular docking and molecular dynamics modelling revealed the stability of the IFI44L-vemurafenib complex, which suggest IFI44L is potential target and vemurafenib could be the better candidate to treat both diseases.

**Abstract:**

Objective: Despite extensive research on periodontitis and rheumatoid arthritis, the underlying molecular connectivity between these condition remains largely unknown. This research aimed to integrate periodontitis and rheumatoid arthritis gene expression profiles to identify interconnecting genes and focus to develop a common lead molecule against these inflammatory conditions. Materials and Methods: Differentially expressed genes (DEGs) of periodontitis and rheumatoid arthritis were identified from the datasets retrieved from the Gene Expression Omnibus database. The network was constructed by merging DEGs, and the interconnecting genes were identified and ranked using GeneMANIA. For the selected top ranked gene, the potential inhibitor was searched using FINDSITE^comb^2.0. Subsequently, the molecular docking and molecular dynamics were performed to determine the binding efficiency and protein-ligand complex stability, respectively. Results: From the network analysis, IFN-induced protein 44-like (IFI44L) was identified as a top ranked gene involved in most of the immunological pathway. With further virtual screening of 6507 molecules, vemurafenib was identified to be the best fit against the IFI44L target. The binding energy and stability of IFI44L with vemurafenib were investigated using molecular docking and molecular dynamics simulation. Docking results show binding energy of −7.7 Kcal/mol, and the simulation results show stability till 100 ns. Conclusions: The identified IFI44L may represent a common drug target for periodontitis and rheumatoid arthritis. Vemurafenib could be a potent anti-inflammatory drug for both diseases.

## 1. Introduction

Periodontitis is an inflammatory polymicrobial disease characterized by gradually destroying supporting tooth structures and bone. If untreated, periodontitis may lead to adult tooth loss [1,2]. The biological mechanism of periodontitis is complex, and the multiple gene interactions and pathways have yet to be deciphered [3,4]. Although a definitive etiology and mechanism has yet to be elucidated, a varied etiology of genetic, immune, environmental, and microbiological factors have been implicated. The existence of certain genetic factors increases the chances of periodontal disease [5,6,7]. A growing body of evidence has linked periodontal disease to various systemic conditions, including heart disease, diabetes, obesity, preterm low birth weight, and rheumatoid arthritis [8,9,10,11,12].

Rheumatoid arthritis (RA) is an autoimmune condition that leads to bone and cartilage degeneration and breakdown. It is a continuous, debilitating, and progressive disease. Common defects, inflammatory joint inflammation, bone resorption, and cartilage destruction are the primary pathophysiological features of RA. The onset and progression of RA may be influenced by genetic factors, inflammation, and immunological dysfunction [13]. The etiology of RA remains unknown despite extensive research into its initiation and progression mechanisms and reasons [14].The latest evidence, supported by twin studies, has discovered that genetic variables play a role in rheumatic disorders [15]. Many individuals can recover if they have been diagnosed and treated properly in the early stage. In subjects with RA, disease-modifying anti-rheumatic drugs (DMARDs) that ameliorate symptoms must be used. Single-use prescriptions are less effective than a combination of biological and synthetic targeted DMARDs [16,17]. Understanding the interplay between immunity, genetics, and the environment is crucial to understanding the causes of RA [18]. Numerous RA risk variants have been discovered in European, Asian, and other ethnic groups in a genome-wide association study (GWAS) [19]. Thousands of differentially expressed genes (DEGs) were tested simultaneously in various experiments on RA with the emergence of high sequencing technologies [20].

Several studies demonstrate remarkable pathological and clinical similarities between RA and periodontitis [21]. At the cellular and molecular level, RA and periodontitis have a lot in common. It is yet unknown how these two inflammatory disorders are linked. Understanding the genetic behavior and its interaction will help to identify core regulatory genes between these diseases that may guide to discover biomarkers and common drug targets. Current advancement in high-throughput technologies facilitates the assessment of thousands of gene expressions at various time scales [22]. Many of these expression datasets have been made freely available through data repositories such as Gene Expression Omnibus and Array Express Archive. Implementing computational analysis on these expression data can help identify novel diagnostic markers and drug target signatures [23]. As a result, it is possible to provide several insights into the biological processes that underpin the disease pathogenesis.

This study aims to add to the knowledge base regarding molecular connectivity between periodontal disease and rheumatoid arthritis. Additionally, our work presents a common drug target between them by analyzing its DEGs network, which enables us to predict a potential inhibitor based on virtual screening and subsequent molecular modeling methods.

## 2. Results

### 2.1. Disease Network

**Figure 1:**
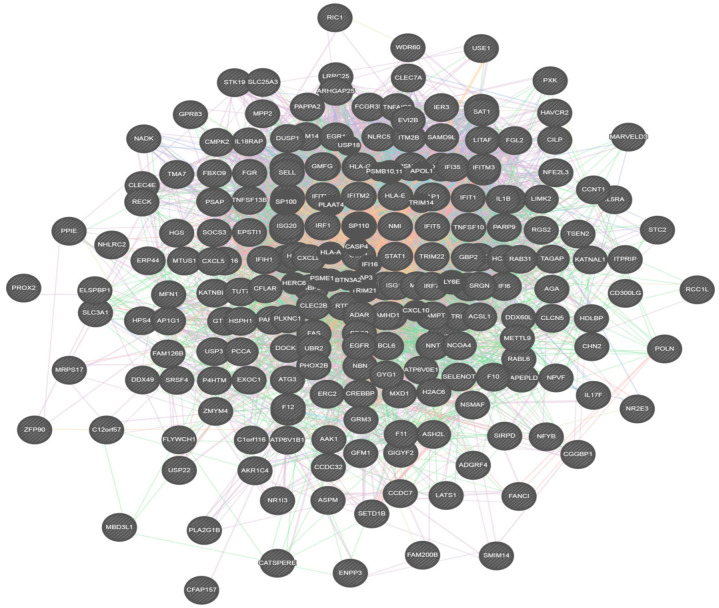
Integrated network of periodontitis and rheumatoid arthritis.

Gene expression datasets for periodontitis (GSE7451) and rheumatoid arthritis (GSE133422) was selected from the GEO database. GEO2R was used to determine two hundred and fifty differentially expressed genes (DEGs) from a dataset of rheumatoid arthritis and periodontitis by comparing with its respective controls available within the datasets. Merging the DEGs of both the conditions, an interaction network was generated (Figure 1) using the Cytoscape software 3.1 version GeneMANIA module. The constructed network presented a multiple molecular connectivity attributed to the DEGs of rheumatoid arthritis and periodontitis, and their functional behavior was demonstrated based on functional enrichment analysis. 

### 2.2. Functional Analysis of Network

To investigate the function of the network, GO enrichment analysis was executed using the DAVID online tool. The enrichment was categorized into biological processes (BP), molecular function (MF) and cellular component (CC). The network enrichment based on biological process showed involvement in type I interferon signaling, cellular response to type I interferon, cytokine-mediated signaling, response to cytokine, immune effector process, cellular response to cytokine stimulus (Supplementary Table S1). Similarly, the molecular functional analysis showed contribution in protein binding, peptide antigen binding, double-stranded RNA binding, 2′-5′-oligoadenylate synthetase activity, and cytokine binding (Supplementary Table S1), respectively. Similarly, the molecules in the network were used to determine their involvement in regulating pathways. The most significantly enriched pathways were related to interferon signaling, cytokine signaling, innate immune system, neutrophil degranulation, interleukin-4 and interleukin-13 signaling and the TRAF3-dependent IRF activation pathway (Supplementary Table S1). Simultaneously, the genesin the network were ranked using GeneMANIA, which demonstrated that IFI44L was the top ranked gene in the network (Table 1).

### 2.3. Virtual Screening and Molecular Docking

**Figure 2:**
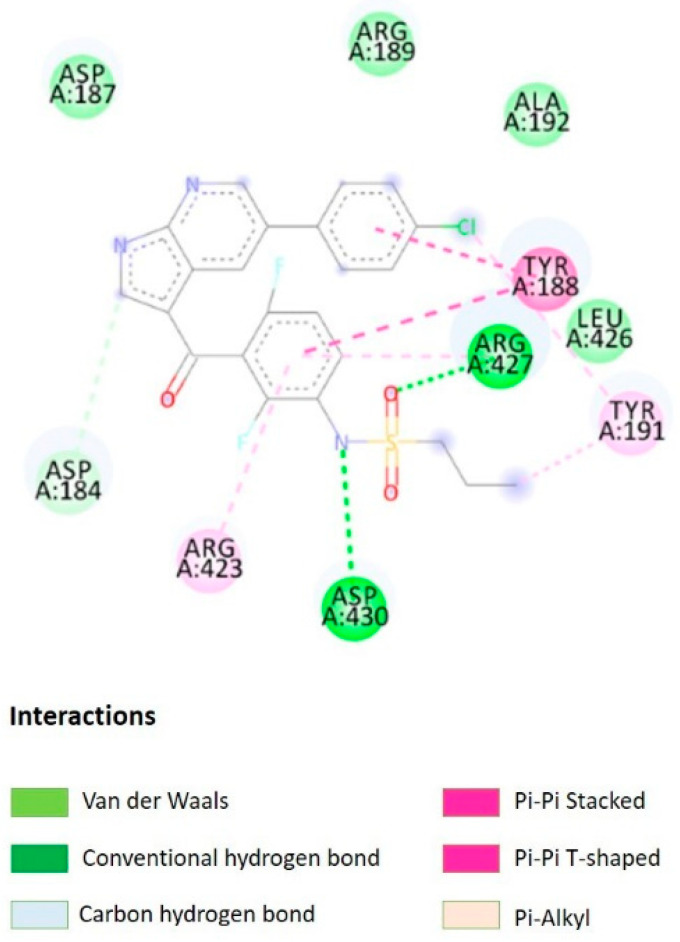
Binding interaction of vemurafenib with IFI144L.

Virtual screening of ligands against IFI44L protein was performed using the FINDSITE^comb^(http://cssb2.biology.gatech.edu/FINDSITE-COMB-II/index_vls.html)(accessed on 12 February 2022). Among the 6507molecules, vemurafenib was found best compound suited against IFI44Lwith the precision score of 0.967031. Further, to test the binding efficiency of the vemurafenib with IFI44L, the molecular docking was performed usingAutoDock Vina. Due to the lack of IFI144L 3D structure in Protein Data Bank, the structure of IFI144L was used to retrieve from the Alpha Fold database. From the 3D structure, water molecules, ions and other hetero-molecules were removed before docking. Following the standard protocol of AutoDock Vina docking, the binding affinity of vemurafenib with IFI44L was found −7.7 Kcal/mol. The interaction between the vemurafenib with IFI44Lprotein was depictedin Figure 2.

### 2.4. Molecular Dynamics Simulation

**Figure 3:**
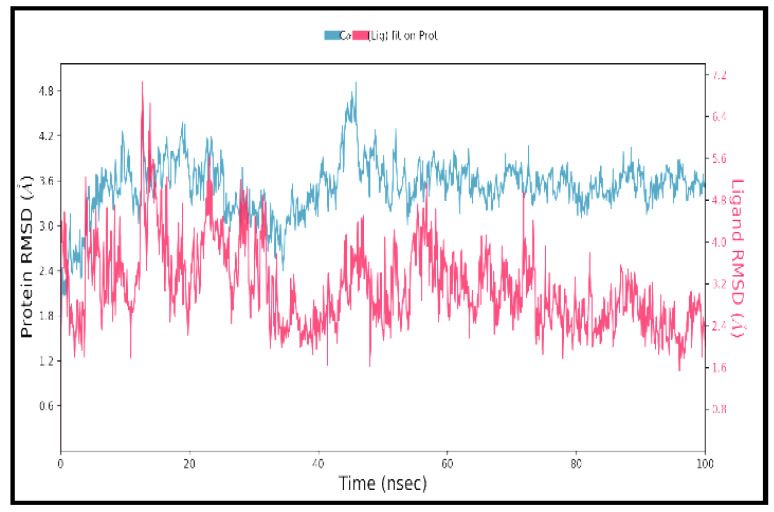
Root mean square deviation (RMSD) of the protein–ligand complex.

**Figure 4:**
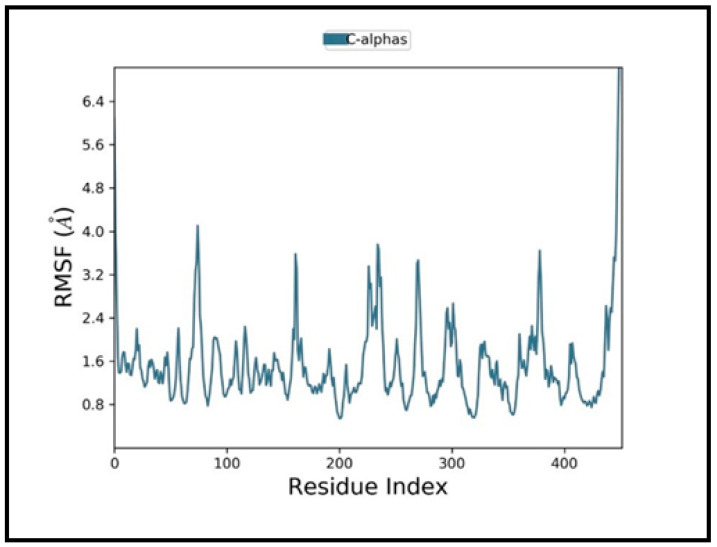
Root mean square fluctuation (RMSF) plot.

**Figure 5:**
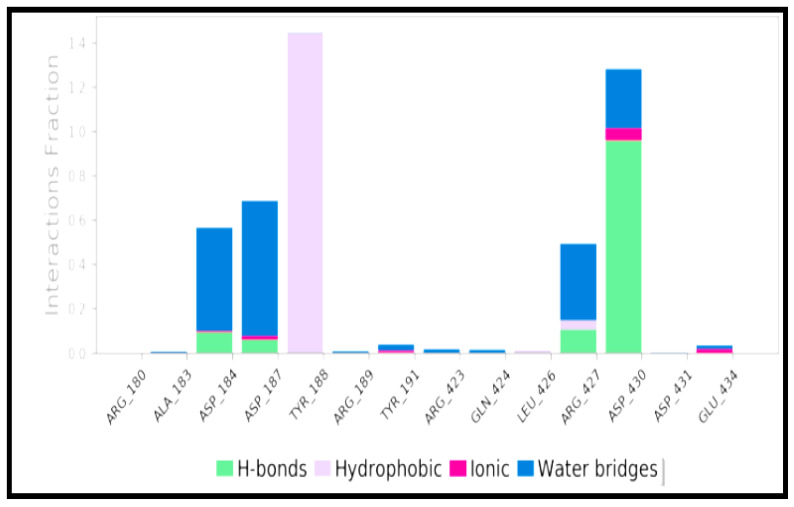
Bar chart displaying the aminoacid contributing to the ligand interaction.

MD simulation is potentially used to assess the dynamic behavior of ligand–protein complexes. Figure 3 depicts the root mean square deviation (RMSD) of the protein-ligand complex over 100ns time. Left *Y*-axis denotes protein RMSD, while the right *Y*-axis denotes ligand RMSD. At 18 ns, the complex achieves stability, and thereafter the RMSD values remain with minimal drift. After equilibrium, the ligand match to protein RMSD values varies around 1.0. These results indicate that the ligands stayed securely attached to the receptor’s binding all through the simulation. Additionally, root mean square fluctuation (RMSF) was assessed, which provides the deviation of a particle on the portions of the structure that fluctuate from the original structure. Lower values denote the stability of the protein–ligand binding. Figure 4 depicts the RMSF of the protein and reveals that the loop areas (=N and C terminal zones) show significant peaks, whereas the α-helices and β-strands appear firmer. Simultaneously, protein–ligand interactions are characterized through hydrogen bonds, ionic interactions, water bridges, and hydrophobic interactions that are visually depicted in Figure 5 in the form of a bar chart. The most significant interactions throughout the docking simulation are hydrogen bonds and hydrophobic interactions (Figure 5). ASP_430 is the most critical bond, and TYR_188 is the most critical hydrophobic interaction.

## 3. Discussion

Expression of genes play a critical role in the pathogenesis of disease, which is increasingly used in medicine to characterize the complicated diseases and aid understanding of the disease mechanism, and development of novel biomarkersand drug targets [24,25,26]. We focused on one of the important problems underpinning the molecular linkage between the periodontitis (PD) and rheumatoid arthritis (RA), which has been widely demonstrated clinically by several common pathogenic and clinical features [27]. Wegner et al. (2010) demonstrated the microbial linkage of *Porphyromonas gingivalis* in both conditions by the presence of peptidylarginine deiminase contributing to RA development [28]. In addition, oral microbiological status causes inflammation that leads to systemic diseases [29]. The backgrounds of these studies encouraged us to look for the molecular evidence connecting both diseases using a complex computation approach. 

Our systematic analysis of network and functional enrichment suggest that most genes in the network were functionally linked to the pathway of innate immunity, the toll-like receptor pathway, and the RIG-like receptor pathway. Among the network genes, IFI44L was ranked highest as the gene that could act as a common target for both of the diseases. IFI44L is a crucial gene connecting rheumatoid arthritis and periodontitis. IFI44L genes play vital roles in inflammatory and immune pathway genes in periodontitis and rheumatoid arthritis. The IFN-induced type 1 gene (IFI44L) promoter DNA methylation level was substantially increased in patients with RA, which is consistent with a previous study in which it was demonstrated thatIFN regulated genes have more robust methylation in patients with RA involvement [30]. Similarly, in periodontitis patients, IFI44LA genes are expressed more extremely (2-fold or greater; n = 149) than in healthy people [31]. Type I IFN levels are usually elevated in tandem, and they are one of the important inflammatory contributors in periodontitis pathogenesis. Considering this outcome, we propose that the elevated IFI44L could be the common drug target between periodontitisand rheumatoid arthritis. Previous studies demonstrate the elevated IFI44L levels across inflammatory conditions, and suggested it to be an efficient antimicrobial target [32,33].

Screening of 6507 known drugs from the DrugBank database showed vemurafenib as a potential ligand against IFI44L based on the deep convolutional neural network method [34]. Vemurafenib is potential drug used for late-stage melanoma [35]. Interestingly, this may have occurred due to the presence of a common regulator between melanoma, periodontal disease and rheumatoid arthritis. There are reports suggesting the association between melanoma with rheumatoid arthritis and periodontal disease [36,37,38]. However, we looked for the binding efficiency of vemurafenib with IFI44L using the molecular docking method. Molecular docking has become an invaluable method in modern drug research and discovery. It can be used to screen, simulate and examine interactions at an atomic level to predict the behavior of molecular processes [39]. The molecular docking shows a binding energy of −7.7 Kcal/mol between vemurafenib andIFI44L. The obtained affinity was significantly better, based on molecular interaction with the surrounding aminoacids.

Finally, molecular dynamics modeling was used to investigate their stability in the simulation environment. The findings revealed that the potential energy of vemurafenib gradually stabilises over time based on the RMSD and RMSF plots. From the plot, it is clear that vemurafenib interact with IFI44L and their complexes are stable in the simulated environment. According to this finding, vemurafenib might be useful for inflammatory diseases like rheumatoid arthritis and periodontal disease. To our knowledge, this study is novel in that it uses an integrative approach towards periodontitis and rheumatoid arthritis for drug target discovery with no experimental data currently available. However, our extensive insilico approach proves that vemurafenib can act as an anti-inflammatory drug by targeting IFN-induced protein 44, and can be a proposed as a potential drug for diseases through the drug repurposing approach.

## 4. Materials and Methods

### 4.1. Dataset and Expression Analysis

The gene expression datasets were collected from the (http://www.ncbi.nlm.nih.gov/geo/) GEO database (accessed on 10 February 2022). Upon extensive searching using key terms related to the study objective, GSE133422 and GSE7451 were selected for our analysis. GSE133422 demonstrate the gene expression of human gingiva epithelial cells exposed to pathogenic biofilms. Similarly, GSE7451 provide the gene expression of patients with primary Sojgren’s syndrome, a condition related to rheumatoid arthritis. Using GEO2R, which is an online R-based tool, the DEGs in each condition were identified by comparing against the control available within the datasets. The DEGs were extracted with the cut-off criterion of logFC > 2 and adj *p*-value set at *p* < 0.05.

### 4.2. Network Construction and Functional Analysis

The Cytoscape GeneMANIA (University of Toronto, Ontario, ON, Canada) module was used to construct the integrated network of periodontitis and rheumatoid arthritis based on DEGs. Additionally, GeneMANIA support ranking of genes in the network to identify the potential genes that link both the conditions, contributing to the common drug target. Afunctional enrichment was carried out for molecules in the merged network using Database for Annotation Visualization and Interactive Discovery (DAVID, Frederick National Laboratory for Cancer Research, Washington, MD, USA). The functional enrichment was classified into biological process and molecular function. Additionally, REACTOME pathway enrichment was executed, whichconveys the involvement of a network molecule in the molecular signaling and metabolic pathways.

### 4.3. Virtual Screening and Molecular Docking

The FINDSITEcomb tool was used for drug screening against the identified top ranked gene(IFI44L) in the network. FINDSITEcomb receives a target sequence as input and applies the convolution neural network (CNN) algorithm to determine the best molecule from the 6507 molecules available in the DrugBank library. Further, the screened ligand was subjected to molecular docking to determine the binding efficiency with the target (IFI44L). For docking, the structure of vemurafenib (Compound CID: 42611257) was retrieved in SDF format from the PubChem database (https://pubchem.ncbi.nlm.nih.gov/) (accessed on 15 February 2022). Similarly, the 3D structure of protein was searched using a relevant protein name or uniprot id (Q53G44) in the PDB database. If there was unavailability of protein structure in PDB, we used a similar search term to collect the predicted structure from the Alphafold database. Using AutoDock Vina (Scripps Research, California, CA, USA), vemurafenib was docked with the IFI44Lto determine the binding efficiency, and their interaction was visualized using the BIOVIA Discovery Studio Visualizer.

### 4.4. Molecular Dynamic Simulation

We used the Desmond (Schrödinger LLC, New York, NY, USA) setup for the high-speed molecular dynamics simulation for the analysis of our model. In general, MD simulations estimate atom movements over time by incorporating Newton’s classical equation of motion. The ligand-binding position in the physiological context was estimated using simulations [40,41]. In our study, the molecular dynamics were carried out for a period of 100 nanoseconds [42]. Protein Preparation Wizard and Maestro (chrödinger LLC, New York, NY, USA) were utilized to preprocess the protein–ligand complex. All of the systems were created using the System Builder tool. TIP3P, an orthorhombic box solvent model, was chosen (Intermolecular Interaction Potential 3 Points Transferable).The OPLS 2005 force field was included in the simulation [43]. Counter ions were utilized to balance the model. 0.15 M sodium chloride (NaCl) was introduced to simulate physiological circumstances. An isobaric-isothermal ensemble was chosen for the phase with a temperature of 300 kelvin and 1 atmospheric pressure maintained throughout. Finally, the stability of the simulation was assessed using root mean square deviation (RMSD), root mean square fluctuation (RMSF), and a histogram of protein–ligand interaction.

## 5. Conclusions

In conclusion, several differentially expressed genes are involved in functions and signaling pathways in rheumatoid arthritis and periodontitis, contributing to their progression and development. Our developed integrated network showed several common linkages between both diseases. Additionally, our network provides IFI44Las a common therapeutic target that can be inhibited by vemurafenib on virtual screening and molecular docking. However, further cell line studies are needed to confirm results. These findings extend our knowledge of these two inflammatory diseases and can pave the way for future studies on drug discovery.

## Figures and Tables

**Table 1:** Top ranked genes extracted from the network constructed from rheumatoid arthritis and periodontitis.

Gene Symbol	Description	Rank
IF44L	Interferon-induced protein 44-like	1
MX1	Mxdynamin-like GTPase 1	2
OAS2	2’-5’-Oligoadenylate synthetase 2	3
MX2	Mxdynamin-like GTPase 2	4
IFI35	Interferon-induced protein 35	5
IRF9	Interferon regulatory factor 9	6
UBE2L6	Ubiquitin conjugating enzyme E2 L6	7
IFIT5	Interferon-induced protein with tetratricopeptide repeats 5	8
IFIT1	Interferon-induced transmembrane protein 1	9
EIF2AK2	Eukaryotic translation initiation factor 2 alpha kinase 2	10

## Supplementary Materials

The following supporting information can be downloaded at: https://www.mdpi.com/article/10.3390/molecules27092749/s1, Table S1: Functional enrichment of the Network.
