# Supplementary material for: Gene Interaction Network Analysis Reveals IFI44L as a Drug Target in Rheumatoid Arthritis and Periodontitis
Source: Molecules. 2022 Apr 25;27(9):2749. doi: 10.3390/molecules27092749 (PMC9104995; doi:10.3390/molecules27092749)
Supplement: Supplementary file 1 [file molecules-27-02749-s001.zip › molecules-1682827-supplementary.pdf]

Table S1. Functional enrichment of the Network

**Biological Process**
Type I interferon signaling pathway
Cellular response to type I interferon
Response to type I interferon
Cytokine-mediated signaling pathway
Response to cytokine
Immune effector process
Cellular response to cytokine stimulus
Defense response to virus
Immune response
Response to virus
Defense response
Innate immune response
Immune system process
Cellular response to organic substance
Response to organic substance
Defense response to other organism
Response to other organism
Response to external biotic stimulus
Response to biotic stimulus
Response to external stimulus
Multi-organism process
Interspecies interaction between organisms
Response to stress
Multi-organism cellular process
Cellular response to chemical stimulus
Symbiosis, encompassing mutualism through parasitism
Regulation of innate immune response
Viral process
Regulation of defense response
Regulation of immune system process
Response to interferon-gamma
Response to stimulus
Regulation of immune response
Regulation of viral genome replication
Interferon-gamma-mediated signaling pathway
Regulation of multi-organism process
Cellular response to stimulus
Positive regulation of defense response
Regulation of response to stimulus
Cell surface receptor signaling pathway
Response to chemical
Regulation of viral process
Regulation of cytokine production
Regulation of symbiosis, encompassing mutualism through parasitism
Viral genome replication
Signal transduction
Cellular response to interferon-gamma
Regulation of response to stress

Cytokine production
Negative regulation of viral genome replication
Response to interferon-beta
Signaling
Regulation of viral life cycle
Cell communication
Negative regulation of multi-organism process
Positive regulation of cytokine production
Single organism signaling
Negative regulation of viral process
Positive regulation of innate immune response
Positive regulation of immune system process
Leukocyte activation
Positive regulation of cellular process
Cell activation
Regulation of biological process
Leukocyte mediated immunity
Negative regulation of biological process
Positive regulation of biological process
Negative regulation of viral life cycle
Regulation of cellular process
Regulation of cell communication
Regulation of signaling
Response to interferon-alpha
Regulation of response to cytokine stimulus
Positive regulation of immune response
Negative regulation of innate immune response
Regulation of catalytic activity
Regulation of signal transduction
Leukocyte activation involved in immune response
Cell activation involved in immune response
Negative regulation of response to stimulus
Neutrophil activation
Regulation of cytokine-mediated signaling pathway
Granulocyte activation
Biological regulation
Myeloid leukocyte activation
Type I interferon production
Regulation of response to external stimulus
Negative regulation of type I interferon-mediated signaling pathway
Regulation of type I interferon production
Neutrophil mediated immunity
Myeloid leukocyte mediated immunity
Regulation of molecular function
Regulation of adaptive immune response
Regulation of type I interferon-mediated signaling pathway
Negative regulation of cellular process
Cellular response to interferon-beta
Positive regulation of response to stimulus
Regulation of multicellular organismal process

Leukocyte degranulation
Response to bacterium
Negative regulation of signal transduction
Neutrophil degranulation
Inflammatory response
Neutrophil activation involved in immune response
Positive regulation of inflammatory response
Myeloid cell activation involved in immune response
Negative regulation of cell communication
Negative regulation of signaling
Positive regulation of type I interferon production
Secretion by cell
Immune system development
Viral life cycle
Regulation of leukocyte mediated immunity
Single-organism process
Activation of innate immune response
Regulation of immune effector process
Positive regulation of response to external stimulus
Exocytosis
Negative regulation of multicellular organismal process
Vesicle-mediated transport
Cellular response to oxygen-containing compound
Positive regulation of leukocyte mediated immunity
Regulation of apoptotic process
Regulation of response to biotic stimulus
Positive regulation of immune effector process
Negative regulation of immune response
Programmed cell death
Positive regulation of chemokine production
Positive regulation of interferon-beta production
Regulation of programmed cell death
Positive regulation of interleukin-1 production
Negative regulation of immune system process
Innate immune response-activating signal transduction
Regulation of hemopoiesis
Regulated exocytosis
Secretion
Antigen processing and presentation of endogenous peptide antigen via MHC class I
Apoptotic process
Pattern recognition receptor signaling pathway
Response to oxygen-containing compound
Regulation of intracellular signal transduction
Positive regulation of multicellular organismal process
Response to nitrogen compound
Regulation of natural killer cell mediated cytotoxicity
Negative regulation of defense response
Cell death
Antigen processing and presentation of endogenous peptide antigen

Regulation of adaptive immune response based on somatic recombination of immune receptors built from Immunoglobulin superfamily domains
Regulation of lymphocyte mediated immunity
Interleukin-1 production
Regulation of interleukin-1 production
Regulation of natural killer cell mediated immunity
Response to endogenous stimulus
Negative regulation of cytokine-mediated signaling pathway
Regulation of hydrolase activity
Intracellular signal transduction
Response to insulin
Negative regulation of response to cytokine stimulus
Interferon-beta production
Single-organism localization
Response to hormone
Antigen processing and presentation of endogenous antigen
Regulation of interferon-beta production
Regulation of cell death
Chemokine production
Hemopoiesis
Regulation of chemokine production
T cell activation
Interleukin-1 beta production
Antigen processing and presentation of endogenous peptide antigen via MHC class I via ER pathway, TAP-Independent
Antigen processing and presentation of endogenous peptide antigen via MHC class I via ER pathway
Response to organonitrogen compound
Cellular response to stress
Interferon-alpha production
Regulation of interferon-alpha production
Cellular response to biotic stimulus
Positive regulation of interleukin-1 beta production
Cellular response to nitrogen compound
Regulation of phagocytosis
Lymphocyte activation
Positive regulation of multi-organism process
Regulation of interleukin-2 production
Positive regulation of tumor necrosis factor superfamily cytokine production
Cellular response to abiotic stimulus
Antigen processing and presentation of exogenous peptide antigen via MHC class I, TAP-independent
Regulation of cell killing
Regulation of interleukin-1 beta production
Hematopoietic or lymphoid organ development
Localization
Response to peptide hormone
Single-multicellular organism process
Cellular response to endogenous stimulus
Natural killer cell mediated cytotoxicity
Defense response to Gram-positive bacterium

Positive regulation of adaptive immune response based on somatic recombination of immune receptors built from Immunoglobulin superfamily domains
Regulation of biological quality
Positive regulation of lymphocyte mediated immunity
Cellular response to lipopolysaccharide
Cell proliferation
Regulation of defense response to virus
Positive regulation of NF-kappab transcription factor activity
Regulation of lymphocyte activation
Response to peptide
Positive regulation of adaptive immune response
Natural killer cell mediated immunity
Antigen processing and presentation of exogenous peptide antigen via MHC class I, TAP-dependent
Regulation of inflammatory response
Cellular response to tumor necrosis factor
Positive regulation of cell killing
Cellular response to insulin stimulus
Regulation of kinase activity
Cellular response to molecule of bacterial origin
Single-organism transport
Regulation of leukocyte activation
Cellular response to oxygen levels
Response to organic cyclic compound
Antigen processing and presentation of exogenous peptide antigen via MHC class I
Regulation of natural killer cell activation
Protein phosphorylation
Regulation of protein phosphorylation
Cellular response to interferon-alpha
Regulation of developmental process
Cellular response to mechanical stimulus
Regulation of cell proliferation
Positive regulation of metabolic process
Cellular response to virus
Regulation of leukocyte proliferation
Positive regulation of cellular metabolic process
Regulation of leukocyte mediated cytotoxicity
Positive regulation of I-kappab kinase/NF-kappab signaling
Cellular response to hormone stimulus
Activation of immune response
Immune response-activating signal transduction
Interleukin-27-mediated signaling pathway
Phosphorylation
Response to tumor necrosis factor
Regulation of protein modification process
Positive regulation of monocyte chemotactic protein-1 production
Leukocyte proliferation
Negative regulation of apoptotic process
Negative regulation of type I interferon production
Regulation of myeloid cell differentiation
Phosphate-containing compound metabolic process

Regulation of cell activation
Cellular response to organonitrogen compound
Regulation of I-kappab kinase/NF-kappab signaling
Positive regulation of macromolecule metabolic process
Regulation of phosphorylation
Lymphocyte mediated immunity
Negative regulation of cell proliferation
Response to oxygen levels
Negative regulation of programmed cell death
Regulation of localization
Regulation of cytokine production involved in immune response
Cytokine production involved in immune response
Immune response-regulating signaling pathway
Adaptive immune response based on somatic recombination of immune receptors built from immunoglobulin Superfamily domains
Negative regulation of natural killer cell mediated cytotoxicity
Antigen processing and presentation of peptide antigen via MHC class I
Leukocyte differentiation
Positive regulation of leukocyte proliferation
Negative regulation of lymphocyte mediated immunity
I-kappab kinase/NF-kappab signaling
Neutrophil chemotaxis
Positive regulation of tumor necrosis factor production
Negative regulation of natural killer cell mediated immunity
Cellular response to lipid
Regulation of T cell activation
Cellular response to decreased oxygen levels
Negative regulation of developmental process
Leukocyte cell-cell adhesion
Phosphorus metabolic process
Cell proliferation involved in kidney development
Positive regulation of T cell mediated immunity
Negative regulation of intracellular signal transduction
Response to mechanical stimulus
Tumor necrosis factor-mediated signaling pathway
Positive regulation of cell communication
Negative regulation of lymphocyte activation
Myeloid leukocyte migration
Cell cycle arrest
Positive regulation of leukocyte mediated cytotoxicity
Positive regulation of signal transduction
Positive regulation of signaling
Regulation of monocyte chemotactic protein-1 production
Monocyte chemotactic protein-1 production
Positive regulation of cell death
Regulation of MAPK cascade
Regulation of transferase activity
Regulation of production of molecular mediator of immune response
Regulation of leukocyte differentiation
Regulation of protein serine/threonine kinase activity

Response to lipid
Cell killing
Lymphocyte proliferation
Positive regulation of leukocyte activation
Positive regulation of T cell activation
Interleukin-2 production
Positive regulation of biosynthetic process
Regulation of lymphocyte proliferation
Positive regulation of apoptotic process
Negative regulation of cytokine production
Mitochondrial membrane fusion
Mononuclear cell proliferation
Positive regulation of response to cytokine stimulus
Response to abiotic stimulus
Positive regulation of interferon-alpha production
Regulation of interleukin-6 production
Antigen processing and presentation
Regulation of mononuclear cell proliferation
Negative regulation of metabolic process
Antigen processing and presentation of exogenous peptide antigen
Defense response to bacterium
Production of molecular mediator of immune response
Positive regulation of programmed cell death
Regulation of protein kinase activity
Positive regulation of homeostatic process
Negative regulation of leukocyte mediated immunity
Regulation of cellular metabolic process
Negative regulation of leukocyte mediated cytotoxicity
Positive regulation of reactive oxygen species metabolic process
Leukocyte chemotaxis
Multicellular organismal process
Response to decreased oxygen levels
Positive regulation of catalytic activity
Regulation of phosphate metabolic process
Regulation of response to interferon-gamma
Regulation of interferon-gamma-mediated signaling pathway
Positive regulation of production of molecular mediator of immune response
Regulation of phosphorus metabolic process
MAPK cascade
Signal transduction by protein phosphorylation
Antigen processing and presentation of exogenous antigen
Cellular protein modification process
Protein modification process
Positive regulation of cell activation
Regulation of macromolecule metabolic process
Positive regulation of cytokine production involved in immune response
Cellular response to organic cyclic compound
Cellular response to interleukin-1
Positive regulation of lymphocyte activation
Positive regulation of gene expression

Positive regulation of cellular biosynthetic process
Regulation of tumor necrosis factor superfamily cytokine production
Tumor necrosis factor superfamily cytokine production
Regulation of leukocyte cell-cell adhesion
Negative regulation of macromolecule metabolic process
Positive regulation of viral process
Blood coagulation, fibrin clot formation
Positive regulation of natural killer cell mediated cytotoxicity
Neutrophil migration
Positive regulation of nitrogen compound metabolic process
Positive regulation of transcription from RNA polymerase II promoter
Regulation of MAP kinase activity
Positive regulation of leukocyte cell-cell adhesion
Negative regulation of cell death
Positive regulation of secretion
T cell differentiation
Negative regulation of cell killing
Positive regulation of cytokine production involved in inflammatory response
Natural killer cell tolerance induction
Leukocyte mediated cytotoxicity
Cellular response to peptide
Antigen processing and presentation of peptide antigen
Positive regulation of acute inflammatory response
Tolerance induction
Positive regulation of cell proliferation
Granulocyte chemotaxis
Transport
Somatic diversification of immune receptors
Negative regulation of leukocyte activation
Positive regulation of hydrolase activity
Positive regulation of RNA metabolic process
Cellular response to growth factor stimulus
Positive regulation of macromolecule biosynthetic process
Cellular response to peptide hormone stimulus
Lymphocyte activation involved in immune response
Positive regulation of transcription, DNA-templated
Positive regulation of nucleic acid-templated transcription
Negative regulation of response to external stimulus
Positive regulation of interferon-gamma production
T cell proliferation
Establishment of localization
Positive regulation of molecular function
Cellular response to dsrna
Regulation of lipid metabolic process
Positive regulation of natural killer cell mediated immunity
Hydrogen ion transmembrane transport
Positive regulation of phosphorylation
Positive regulation of RNA biosynthetic process
Female pregnancy
Regulation of myd88-dependent toll-like receptor signaling pathway

Activation of phospholipase A2 activity
Positive regulation of fibrinolysis
Regulation of cell differentiation
Cytoplasmic pattern recognition receptor signaling pathway in response to virus
Transcription from RNA polymerase II promoter
Negative regulation of immune effector process
Macromolecule modification
Negative regulation of catalytic activity
Positive regulation of sequence-specific DNA binding transcription factor activity
Regulation of metabolic process
Homeostasis of number of cells
Adaptive immune response
Regulation of secretion by cell
Response to growth factor
Response to organophosphorus
Positive regulation of lymphocyte proliferation
Cellular response to external stimulus
Regulation of reactive oxygen species metabolic process
Cellular response to hypoxia
Myeloid cell differentiation
Positive regulation of protein phosphorylation
Response to lipopolysaccharide
Positive regulation of mononuclear cell proliferation
Response to interleukin-1
Negative regulation of molecular function
Regulation of T cell mediated immunity
Negative regulation of cell differentiation
Negative regulation of protein phosphorylation
Regulation of endocytosis
Positive regulation of secretion by cell
Negative regulation of cell activation
Regulation of primary metabolic process
Positive regulation of nucleobase-containing compound metabolic process
Positive regulation of viral genome replication
Response to hypoxia
Homeostatic process
Granulocyte migration
Negative regulation of nucleobase-containing compound metabolic process
Regulation of cell adhesion
Positive regulation of cell-cell adhesion
Regulation of nucleobase-containing compound metabolic process
Temperature homeostasis
Negative regulation of extrinsic apoptotic signaling pathway via death domain receptors
Regulation of sequence-specific DNA binding transcription factor activity
Negative regulation of cell cycle
Response to molecule of bacterial origin
Proton transport
Hydrogen transport
Natural killer cell activation
Protection from natural killer cell mediated cytotoxicity

Positive regulation of MHC class I biosynthetic process
Positive regulation of glomerular mesangial cell proliferation
Response to purine-containing compound
Alpha-beta T cell activation
Negative regulation of cellular metabolic process
Regulation of RNA metabolic process
Toll-like receptor signaling pathway
Negative regulation of cell adhesion
Macromolecule metabolic process
Lymphocyte differentiation
Multi-multicellular organism process
Interleukin-6 production
Single organism reproductive process
Single-organism cellular process
Positive regulation of interleukin-6 production
Interaction with host
Antimicrobial humoral response
Regulation of stress-activated MAPK cascade
Positive regulation of phospholipase A2 activity
MHC class I biosynthetic process
Superoxide anion generation
Regulation of viral entry into host cell
Positive regulation of protein modification process
Regulation of cellular catabolic process
Regulation of interleukin-8 production
Phagosome maturation
Regulation of stress-activated protein kinase signaling cascade
Regulation of cysteine-type endopeptidase activity
Regulation of ERK1 and ERK2 cascade
Cell chemotaxis
Positive regulation of T cell proliferation
Regulation of catabolic process
Tumor necrosis factor production
Nucleobase-containing compound biosynthetic process
Regulation of erythrocyte differentiation
Regulation of alpha-beta T cell activation
Positive regulation of phosphorus metabolic process
Positive regulation of phosphate metabolic process
Regulation of secretion
Negative regulation of phosphorylation
Negative regulation of T cell differentiation
Negative regulation of extrinsic apoptotic signaling pathway
Regulation of protein localization
Endocytosis
Positive regulation of cell proliferation involved in kidney development
Regulation of germinal center formation
Regulation of CD8-positive, alpha-beta T cell proliferation
Regulation of MHC class I biosynthetic process
Regulation by virus of viral protein levels in host cell
Regulation of cell-cell adhesion

Negative regulation of leukocyte differentiation
Positive regulation of viral life cycle
DNA metabolic process
Histone modification
Positive regulation of protein serine/threonine kinase activity
Regulation of T cell proliferation
Organelle membrane fusion
Regulation of Notch signaling pathway
Response to carbohydrate
Regulation of cellular response to growth factor stimulus
Negative regulation of leukocyte apoptotic process
Reproductive process
Reproduction
T cell mediated immunity
Regulation of tumor necrosis factor production
Response to testosterone
Regulation of multicellular organismal development
Positive regulation of cell cycle
Response to dsrna
Regulation of ribonuclease activity
NIK/NF-kappab signaling
Positive regulation of intracellular signal transduction
Negative regulation of response to biotic stimulus
Ossification
Covalent chromatin modification
Negative regulation of blood coagulation
Leukocyte apoptotic process
Protein localization
Intracellular receptor signaling pathway
Heterocycle biosynthetic process
Antimicrobial humoral immune response mediated by antimicrobial peptide
Interferon-gamma production
T cell activation involved in immune response
Response to nutrient
Cellular response to radiation
Negative regulation of hemostasis
Negative regulation of transport
Negative regulation of MAPK cascade
Positive regulation of kinase activity
Aromatic compound biosynthetic process
Cellular response to transforming growth factor beta stimulus
Negative regulation of natural killer cell activation
Regulation of glomerular mesangial cell proliferation
Cell proliferation involved in metanephros development
T cell activation via T cell receptor contact with antigen bound to MHC molecule on antigen presenting cell
Regulation of phosphatidylinositol 3-kinase activity
Blood coagulation
Stimulatory C-type lectin receptor signaling pathway
Negative regulation of phosphate metabolic process
Negative regulation of phosphorus metabolic process

Notch signaling pathway
Regulation of kidney development
Embryo implantation
Positive regulation of cytokine-mediated signaling pathway
Regulation of vasculature development
Regulation of interferon-gamma production
Regulation of transport
Regulation of body fluid levels
Negative regulation of RNA metabolic process
Erythrocyte differentiation
Lysosomal transport
Response to transforming growth factor beta
Single organism cell adhesion
Hemostasis
Negative regulation of coagulation
Negative regulation of lymphocyte differentiation
Protein metabolic process
Glomerular mesangial cell proliferation
MDA-5 signaling pathway
Positive regulation of glomerulus development
Positive regulation of cellular component movement
Wound healing
Coagulation
Innate immune response activating cell surface receptor signaling pathway
Regulation of extrinsic apoptotic signaling pathway via death domain receptors
Leukocyte migration
Regulation of cell migration
Response to nutrient levels
Negative regulation of protein modification process
Positive regulation of cellular protein metabolic process
Germinal center formation
Positive regulation of mitochondrial membrane potential
Regulation of memory T cell differentiation
Toll-like receptor 7 signaling pathway
Skeletal muscle cell differentiation
Protein methylation
Protein alkylation
Single organismal cell-cell adhesion
Cellular response to DNA damage stimulus
Regulation of leukocyte chemotaxis
**Molecular Function**
Protein binding
Peptide antigen binding
Identical protein binding
Zinc ion binding
Atp binding
Tap binding
Gtp binding
Double-stranded rna binding

2'-5'-oligoadenylate synthetase activity
Protein kinase inhibitor activity
Cytokine binding
Enzyme binding
Guanyl-nucleotide exchange factor activity
Transcription regulatory region sequence-specific dna binding
Kinase binding
Nucleic acid binding
Dna binding
Receptor binding
Double-stranded dna binding
Bile acid binding
Cysteine-type endopeptidase activity involved in apoptotic signaling pathway
Nucleotide binding
GTPase activity
**Molecular Pathway**
Interferon alpha/beta signaling
Interferon Signaling
Immune System
Cytokine Signaling in Immune system
Interferon gamma signaling
Innate Immune System
Neutrophil degranulation
Antigen Presentation: Folding, assembly and peptide loading of class I MHC
Regulated Necrosis
Endosomal/Vacuolar pathway
Interleukin-4 and Interleukin-13 signaling
TRAF3-dependent IRF activation pathway
Diseases of hemostasis
Defects of contact activation system (CAS) and kallikrein/kinin system (KKS)
FOXO-mediated transcription of cell death genes
TP53 Regulates Transcription of Cell Death Genes
Antiviral mechanism by IFN-stimulated genes
DDX58/IFIH1-mediated induction of interferon-alpha/beta
Immunoregulatory interactions between a Lymphoid and a non-Lymphoid cell
ER-Phagosome pathway
Intrinsic Pathway of Fibrin Clot Formation
Antigen processing-Cross presentation
Pyroptosis
TRAF6 mediated IRF7 activation
Signaling by Interleukins
Negative regulators of DDX58/IFIH1 signaling
Formation of Fibrin Clot (Clotting Cascade)
